# Exploring Experiences of People With Knee Osteoarthritis Who Participated in an Online Unsupervised Tai Chi Program: A Qualitative Study

**DOI:** 10.1002/acr2.70123

**Published:** 2025-11-27

**Authors:** Shiyi Julia Zhu, Travis Haber, Rachel K. Nelligan, Kim L. Bennell, Rana S. Hinman, Belinda J. Lawford

**Affiliations:** ^1^ Centre for Health, Exercise and Sports Medicine The University of Melbourne Melbourne Victoria Australia

## Abstract

**Objective:**

It is unclear how people with osteoarthritis feel about online Tai Chi. This study aimed to explore the experiences of people with knee osteoarthritis who participated in an online unsupervised Tai Chi program.

**Methods:**

A qualitative study nested within a randomized controlled trial was conducted. Semistructured phone interviews were held with 20 participants with knee osteoarthritis who took part in a 12‐week online unsupervised Tai Chi program. Interviews explored participant experiences and were audio recorded, transcribed verbatim, and analyzed thematically using an inductive approach.

**Results:**

Four themes (each with two subthemes) were developed: (1) online unsupervised Tai Chi offers flexibility (the ability to pause, rewind, and repeat facilitates learning; able to practice anytime, anywhere); (2) variable user experience (most found it enjoyable and calming; some found it repetitive and boring); (3) learning challenges and strategies (the lack of feedback can be challenging; practice makes better); and (4) online unsupervised Tai Chi is effective for most but not all (improved outcomes and motivated to be more active; no perceived changes in outcomes).

**Conclusion:**

Most people with knee osteoarthritis reported positive experiences with a 12‐week online unsupervised Tai Chi program. The identified challenges and relevant improvements have the potential to inform modifications and refine the program before its planned public release, with the aim to enhance Tai Chi exercise accessibility and uptake among people with osteoarthritis in the broader community.

## INTRODUCTION

Knee osteoarthritis (OA) is a highly prevalent and debilitating condition, affecting 2.2 million Australians, with a 58% increase in prevalence expected by 2032 relative to 2018 due to the aging population.^1^, ^2^ Exercise is advocated as a first‐line treatment by all current clinical guidelines.^3^, ^4^ However, it remains underused among people with knee OA.^5^, ^6^ Barriers to uptake include cost and poor accessibility of exercise services and programs.^7^ Developing new ways of overcoming these barriers could potentially enhance uptake of exercise and help reduce the burden of knee OA.

**SIGNIFICANCE & INNOVATIONS**

- In this qualitative study using semistructured interviews to explore experiences of people with knee osteoarthritis (OA) participating in an online unsupervised Tai Chi program, most participants appreciated the flexibility of learning Tai Chi online through prerecorded videos and noted physical and mental improvements.
- Some participants found the program repetitive and boring and felt that the lack of feedback negatively impacted their adherence.
- The identified challenges and suggested improvements can inform modifications and refine the program before its planned public release, with the aim to enhance accessibility and uptake of Tai Chi exercise among people with OA in the broader community.

One way in which to overcome accessibility barriers to exercise is by delivering it online. Since the COVID‐19 pandemic, digital exercise programs for OA care have rapidly expanded.^8^, ^9^ These programs are delivered through various methods, such as synchronized videoconferencing calls, asynchronous prerecorded videos, phone‐based sessions, app‐based platforms, or multitechnology approaches.^8^ There is evidence that digitally delivered exercise programs are effective in improving pain and physical function for people with knee OA.^9^ For example, a free resistance exercise program delivered via a self‐directed website (ie, without any clinician supervision) was found to be effective for knee OA,^10^ with most users also reporting positive experiences.^11^ Digital exercise programs can also potentially reduce costs associated with exercise participation.

Tai Chi is a popular form of exercise and is recommended by clinical guidelines for management of knee OA.^3^, ^4^ Tai Chi differs from other forms of exercise, such as resistance exercise, due to its focus on body awareness and mindfulness.^12^ Specifically, Tai Chi integrates the mind, body, and spirit, requiring people to focus on their breathing and the alignment of their body's position and movements, which has been shown to improve postural control, physical function, and stiffness in older adults with knee OA.^13^, ^14^ Tai Chi is thought to contribute to improvements in symptoms by reducing systemic inflammation, improving muscle strength, proprioception, and/or joint stability.^15^, ^16^ Because it is typically practiced in person within a group setting, access to Tai Chi can be challenging and inconvenient, particularly in regional areas where the prevalence of OA is high but Tai Chi classes are limited.^2^ Evidence from non‐OA populations has shown that online or unsupervised Tai Chi interventions can be effective, including for community‐dwelling older adults and people with dementia, cardiovascular conditions, cancers, and other chronic conditions.^17^, ^18^, ^19^, ^20^, ^21^, ^22^, ^23^ However, no studies have evaluated the effectiveness or acceptability of digital unsupervised Tai Chi programs for people with knee OA.

Previously, we developed a 12‐week, evidence‐based unsupervised online Tai Chi intervention housed on the website MyJoint Tai Chi and supported by an exercise adherence app.^24^ The effectiveness of this intervention is currently being evaluated in a randomized controlled trial (RCT) involving 178 people with a clinical diagnosis of knee OA (trial registry number: ACTRN12623000780651).^25^ Given the importance of user acceptability in the successful implementation of health interventions, qualitative research is needed to explore the experiences of individuals participating in this online Tai Chi program.^26^ Thus, the aim of this study was to explore the experiences of people with knee OA who participated in the MyJoint Tai Chi program.

## METHODS

### Study design

This qualitative study was nested within an RCT (trial registry number: ACTRN12623000780651). The design of this qualitative study was based on an interpretivist paradigm. According to this paradigm, knowledge about a phenomenon is developed by gathering perceptions and interpretations of participants who experience it.^27^ A phenomenological framework was used, which focuses on the lived experiences of people involved in the issue being researched.^28^ This study is reported in accordance with the Consolidated Criteria for Reporting Qualitative Research guidelines.^29^ Ethics approval was obtained from the Human Research Ethics Committee of The University of Melbourne on February 21, 2024 (reference number: 2024‐27969‐49792‐3).

### Participant recruitment

Participants in this study composed a subsample of those allocated to the intervention arm of the overarching RCT and who had completed the 12‐week online Tai Chi intervention. Participants were unblinded, as they were aware they might be allocated to an online unsupervised Tai Chi program as part of the trial intervention. In the RCT, participants were recruited from the Australia‐wide community via internet sources (eg, social media, radio, and online newspapers). Eligibility criteria for the RCT have been described in detail.^30^ Briefly, they include the following: a diagnosis of knee OA using the National Institute for Health and Care Excellence clinical OA criteria^31^; history of knee pain for at least three months; knee pain on most days in the past month; knee pain in the past week during walking; ≥4 on an 11‐point numerical rating scale; and home internet connection and a computer/tablet device that enables access to the internet. Exclusion criteria included the following: recent knee surgery (past six months); on the waiting list for/planning knee surgery in the next three months; previous knee joint replacement on the affected side; participating in regular (at least once per week) exercise over the past three months.

Participants were purposively sampled to participate in this qualitative study to ensure variation across age, gender, geographic location (eg, metropolitan, regional), change in symptoms post intervention, satisfaction with the intervention, and likelihood of recommending the Tai Chi program to others. The sample size was not predetermined but dictated by the principle of theoretical code meaning saturation.^32^ This process involved systematically reviewing the interviews to identify distinct codes and then examining subsequent interviews to determine whether new meanings, dimensions, or nuances emerged for each code.^32^, ^33^ Recruitment ceased and saturation was considered achieved when the researchers believed they had obtained a comprehensive understanding of the phenomenon of interest and no substantive new information or themes were emerging from new interviews.^32^, ^33^, ^34^

**Figure 1:**
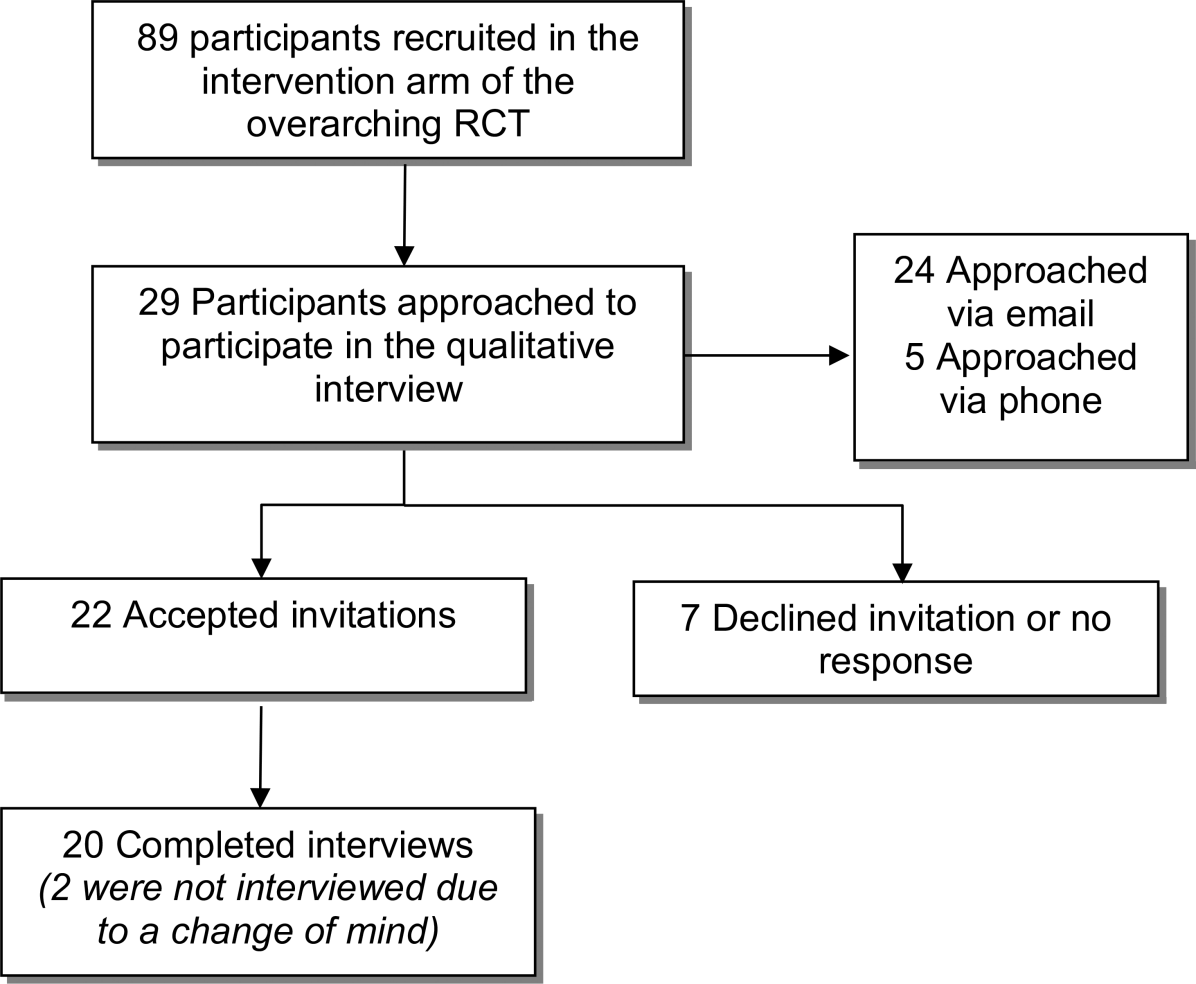
Participants recruitment flowchart. RCT, randomized controlled trial.

Potential participants were recruited by researcher SJZ, who emailed information about the study or phoned with an invitation to participate. Online consent was gained via REDCap. Participants were invited to an interview as they completed outcome measures at 12 weeks. All interviews were conducted within three months of the participant completing the Tai Chi intervention. The participant recruitment flowchart is shown in Figure 1.

### Online unsupervised Tai Chi intervention

**Figure 2:**
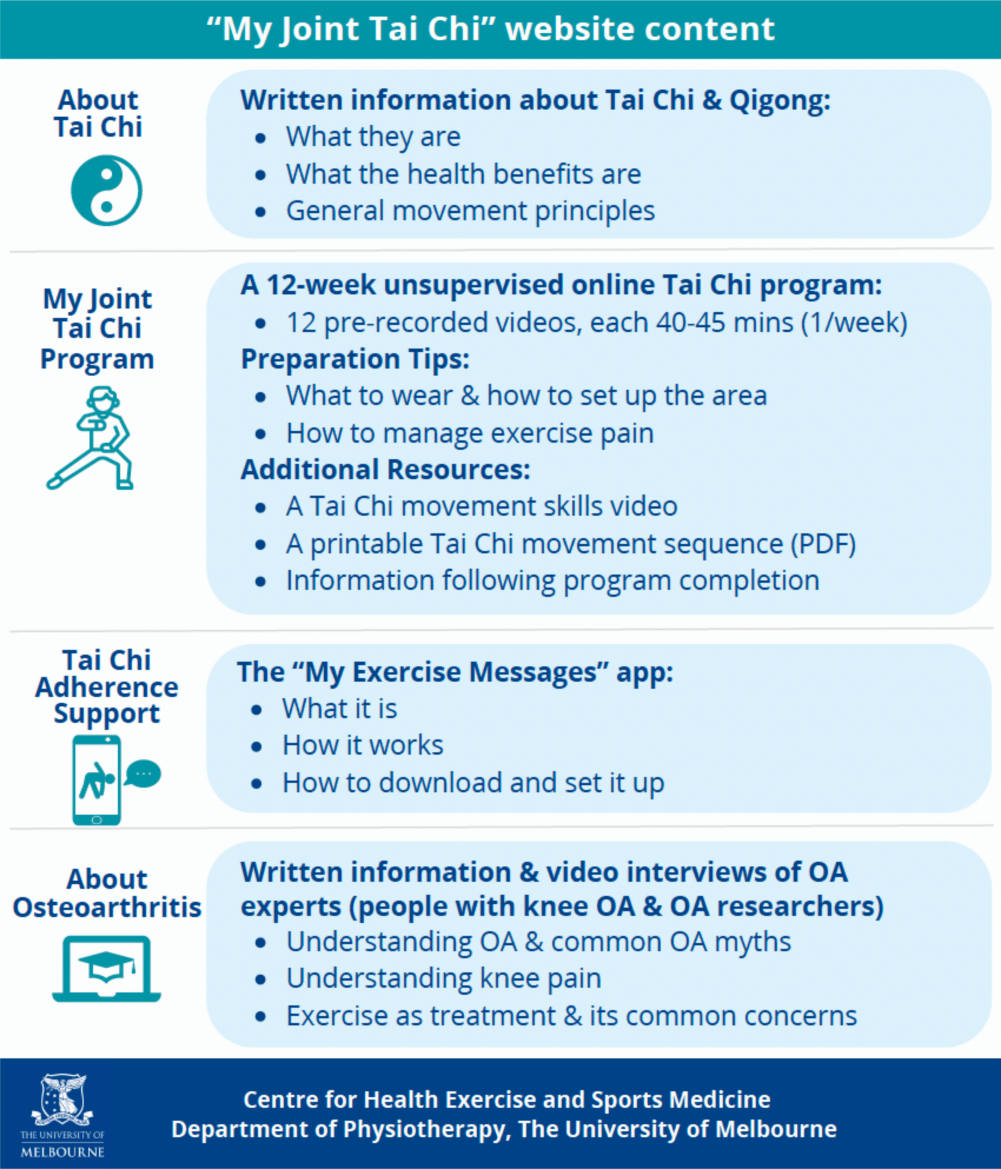
Description of the content in the four discrete sections of the MyJoint Tai Chi website. Reprinted, with permission, from ref 24.

The online unsupervised Tai Chi intervention has been described elsewhere.^24^ Briefly, participants were given access to a bespoke website, MyJoint Tai Chi (https://myjoint-taichi.org/), which contained four sections, described in Figure 2. A detailed description of the website is provided in the Supplementary File. Specifically, the MyJoint Tai Chi program includes 12 prerecorded videos for participants to do across 12 weeks (one video per week). Each week, participants were assigned one video, which they were required to follow three times per week at home at a time of their choosing. Each video (40–45 minutes) began with a warm‐up and ended with a cooldown. The central portion featured a modified 10‐form Yang style Tai Chi, which involves slow and controlled movement, with modifications made for those with lower limb OA and little prior Tai Chi experience. The 12‐week program started with simple Tai Chi movements, with new movements added each week to progress the difficulty. Information on preparation, managing exercise pain, and postprogram recommendations was also provided on the website. Additionally, participants were encouraged to download the My Exercises Messages mobile app,^35^ designed for people with hip and/or knee OA to facilitate exercise adherence and set up in the first week. In the app each week, users were asked to self‐report the number of days they had completed Tai Chi, and those with low adherence (two or fewer sessions per week) were prompted to select a reason from a list of exercise barriers. A tailored message (behavior change technique) was then provided to help them overcome that barrier. Users also received two notifications per week containing messages designed to encourage regular exercise.

### Data collection

Individual interviews were conducted by SJZ (PhD candidate, physiotherapist, and coordinator of the overarching RCT, who had recruited and enrolled all participants and had undergone training in qualitative research). A semistructured interview guide was developed and used to provide a framework for each interview (Table 1), seeking to explore experiences with the Tai Chi intervention. All interviews were conducted via telephone and were audio recorded. Each interview lasted between 20 and 60 minutes. At the beginning of each interview, participants were reassured that there were no right or wrong answers and were encouraged to share their honest experiences with the program. The study information sheet also emphasized that their responses would remain confidential and would not affect their relationship with the researchers. Explicit questions in the interview guide were used to prompt participants to consider negative aspects of the program or challenges they experienced during participation. Further probing questions were asked if participants mentioned any negative aspects. Recordings were transcribed by an external transcription service. All data were deidentified and stored in a password‐protected secure computer file on the university server.

**Table 1:** Semistructured interview guide

**Interview questions**
1. What motived you to join this Tai Chi study?
(a) What experience, if any, have you had with Tai Chi?
(b) When you first read the research ads online, what were your thoughts on this online unsupervised Tai Chi program?
2. Think back when you were doing this 12‐week program. Overall, what did you think of it?
3. Think back to the environment and space when you did the program. Can you describe where did you do the program and how you set up the space?
(a) How did you find using that space to do the program?
(b) How did you find the setup?
(c) Can you describe if anything made it challenging for you to do the program at home?
4. Normally, Tai Chi is done in person in a group setting, but this was a home‐based Tai Chi program—so you needed to follow along with the video and learn the movements online without any instructions or feedback. How did you find learning Tai Chi online in this format without supervision?
(a) What was the overall learning process like for you?
(b) What aspect of the program did you particularly like?
(c) What aspect of the program were you less keen on?
(d) What could have made it better?
*Follow‐up questions based on participant's responses:*
(e) How did you find the pace of the online class?
(f) Would it be the same if it were done in another format?
(g) Was it (*insert participant's response*) because it is delivered online?
(h) If there is a different way to learn Tai Chi, would your experience be different?
(i) What would be an ideal program to you?
5. As you would have found, Tai Chi is repetitive. One of the reasons that it is repetitive is to help you learn the Tai Chi movements so that you can do the sequence without needing to follow an instructor or video. Reflecting on the program, can you describe your ability to do the Tai Chi movement by yourself?
(a) What made it easy or difficult? Why?
(b) What aspect of this online program could have helped you more in learning or practicing Tai Chi?
6. As part of the study, each week there was one new video for you to do, and you were asked to follow along with that video three times that week. What are your thoughts on doing the program three times a week?
(a) What helped you stick to the program?
(b) What made it hard?
(c) What could have helped you (more) in sticking to the program?
7. Following the 12‐week program, what has changed about your health and well‐being?
(a) Can you describe if you have had any changes in your knee condition?
(b) What other changes have you noticed since you started the program? For example, your overall health/physical activity level/sleep/mood
(c) The online Tai Chi instructor demonstrated some elements of mindfulness and meditation in the video, such as mindful breathing, posture alignment, or foot positionings. Have you incorporated any of these Tai Chi learnings into your everyday life?
8. Now that you have finished this Tai Chi program, what do you plan to do next to help manage your knee? Do you plan to continue practicing Tai Chi?
(a) If yes, in what way do you think you will continue Tai Chi practice?
(b) If no, why?
9. Would you recommend this program to family and friends who have knee issues?
(a) Why or why not?
10. Is there anything else you would like to add about the MyJoint Tai Chi program?

Demographic details, such as age, gender, education level, employment status, geographic location (metropolitan or regional), past experience with Tai Chi, and baseline knee pain score, were collected via the baseline questionnaire in the overarching RCT. Other information, such as responses to self‐reported perceived change in symptoms, satisfaction, and likelihood of recommending the Tai Chi program to others, was collected via the RCT follow‐up questionnaire at 12 weeks.

### Data analysis

Reflexive thematic analysis was used for analyzing the interview data. This approach recognizes that researchers actively contribute to developing themes, influenced by their own perspectives and interpretations.^36^ Data were inductively coded following steps based on the work of Braun and Clarke^36^:

1. Two independent researchers, SJZ and TH (both research physiotherapists with training in qualitative methods and prior knowledge of Tai Chi as a therapeutic intervention for knee OA; TH has no involvement in the overarching RCT), familiarized themselves with the interview data by reading and rereading transcripts. Sections of the audio recordings were also listened to, as required.
2. The two researchers then separately conducted open coding by rereading each transcript line by line and applying a “code” to summarize passages of data interpreted as important and relevant to the research question. They subsequently independently grouped codes into subthemes, and these subthemes were grouped into preliminary themes.
3. The two researchers then met to discuss their analysis and impressions from the data and finalize the themes and subthemes.
4. Aiming to enhance credibility, the agreed‐on themes and subthemes were reviewed by a third researcher (BJL, who has no involvement in the RCT), who also read all the transcripts. They confirmed that the themes are credible and rooted in the data.
5. Themes
6. were further reviewed by the broader research team until a consensus was reached to further ensure data credibility and confirmability.

NVivo version 14 software was used to facilitate data coding. Data collection and analysis took place simultaneously. Exemplary quotes were deidentified to ensure confidentiality.

## RESULTS

### Demographics

Interviews were conducted with 20 participants who participated in online unsupervised Tai Chi. Participant characteristics are presented in Table 2. On average, participants were 62 years old (SD 7); the majority were female (n = 16; 80%), and baseline knee pain during walking was 6 out of 10 (SD 1). Only one participant had past experience with Tai Chi. Fifteen participants (75%) reported improvements in their knee condition and were adherent to the program. Overall, participants were generally satisfied with the program, giving it an average rating of 8 out of 10 (SD 3; higher scores indicate higher levels of satisfaction).

**Table 2:** Participant characteristics (N = 20)*

No.	Age, y	Gender	Geographic location^a^	Highest education level	Employed^b^	Past experience with Tai Chi^c^	Baseline knee pain^d^	Perceived overall change in knee condition^e^	Program satisfaction^f^	Program recommendation likelihood^g^	Adherence^h^
1	68	M	Major city	Secondary school	No	No	6	Moderately improved	7	8	Nonadherent
2	66	F	Major city	University or tertiary institute	No	No	5	Moderately improved	8	7	Adherent
3	68	M	Major city	University or tertiary institute	Yes	No	7	Same	8	8	Adherent
4	74	F	Major city	University or tertiary institute	No	No	6	Moderately improved	8	8	Adherent
5	61	F	Outer regional	University or tertiary institute	Yes	No	5	Markedly improved	10	10	Adherent
6	62	M	Major city	University or tertiary institute	Yes	No	4	Markedly improved	10	10	Adherent
7	55	Nonbinary	Major city	Secondary school	No	No	7	Same	6	7	Nonadherent
8	54	F	Inner regional	University or tertiary institute	Yes	No	6	Markedly improved	9	9	Adherent
9	62	F	Major city	Secondary school	Yes	No	6	Same	0	0	Nonadherent
10	54	F	Major city	University or tertiary institute	Yes	Yes	7	Moderately improved	10	10	Adherent
11	50	F	Major city	University or tertiary institute	Yes	No	7	Markedly improved	8	8	Nonadherent
12	59	F	Inner regional	Higher university degree	Yes	No	5	Moderately improved	7	7	Adherent
13	53	F	Inner regional	University or tertiary institute	Yes	No	5	Markedly improved	10	10	Adherent
14	68	F	Inner regional	University or tertiary institute	No	No	4	Markedly improved	10	10	Adherent
15	70	F	Major city	University or tertiary institute	No	No	5	Markedly improved	10	10	Adherent
16	61	F	Major city	University or tertiary institute	Yes	No	7	Moderately improved	9	10	Adherent
17	62	F	Major city	Secondary school	No	No	8	Same	3	5	Nonadherent
18	57	F	Inner regional	Higher university degree	Yes	No	6	Moderately improved	7	8	Adherent
19	68	F	Inner regional	University or tertiary institute	No	No	5	Markedly improved	10	10	Adherent
20	59	F	Major city	University or tertiary institute	Yes	No	7	Moderately worsened	8	8	Adherent
Mean (SD)	62 (7)	n/a	n/a	n/a	n/a	n/a	6 (1)	n/a	8 (3)	8 (2)	n/a

* F, female; M, male; n/a, not applicable.

^a^ Geographic location classification was based on the Australian Statistical Geography Standard Remoteness Structure: major cities, inner regional, outer regional, remote, and very remote.^47^

^b^ Employed on a casual, part‐time, or full‐time basis.

^c^ Past experience with Tai Chi was assessed via the question “Have you undertaken any Tai Chi practice in the past 2 years?” with response options “yes” or “no.”

^d^ Baseline knee pain during walking in the past week was assessed via an 11‐point numerical rating scale from 0 = “no pain” to 10 = “worst pain possible.”

^e^ Perceived global rating of overall change in knee condition was assessed using the following categories: “markedly improved,” “moderately improved,” “same,” “moderately worsened,” and “markedly worsened.” Participants who indicate that were “markedly improved” or “moderately improved” were classified as improved, and all others were classified as not improved.

^f^ Program satisfaction was assessed on an 11‐point numerical rating scale, where 0 = “not at all satisfied,” and 10 = “extremely satisfied.”

^g^ Program recommendation likelihood was assessed on an 11‐point numerical rating scale, where 0 = “not at all likely,” and 10 = “extremely likely.”

^h^ Adherence was assessed using the self‐reported number of days per week MyJoint Tai Chi was performed, averaged from data collected at 2 weeks and at 12 weeks. Participants were classified as “adherent” if they report doing the program on 2 or more days per week, and all other participants were classified as “nonadherent.”

### Thematic analysis

Four themes (each with two subthemes) were developed: (1) online unsupervised Tai Chi offers flexibility (the ability to pause, rewind, and repeat facilitates learning; able to practice anytime, anywhere); (2) variable user experience (most found it enjoyable and calming; some found it repetitive and boring); (3) learning challenges and strategies (the lack of feedback can be challenging; practice makes better); and (4) online unsupervised Tai Chi is effective for most but not all (improved outcomes and motivated to be more active; no perceived changes in outcomes). A summary of themes and subthemes are presented in the following sections. The exemplary quotes are presented in Table 3.

**Table 3:** Themes, subthemes, and exemplary quotes from the patient interviews

	Quotes
Theme 1. Online unsupervised Tai Chi offers flexibility	
1.1 The ability to pause, rewind, and repeat facilitates learning	“I can pause and I can rewind, go back and redo different sections. I don't have to just keep going. If I'm not quite sure what's happening, I can just go back and go, ‘Oh yes, OK, now I understand it and keep moving forward.’” P4
	“It wasn't that easy. But what I just done after week six, it become easier, but I would still stop it, wind it back three, four, five minutes and watch it again, watch she is doing. And it helped me immense to learn.” P6
	“If I was stuck and I hadn't got the move, I would just pause and go back, rewind and repeat it until I felt that I'd understood it. So that was good, being able to go at my own pace and then recap if I needed to.” P8
	“You can't do that in an (in person) class because you kind of feel quite pressured in a class to keep moving through. But with this one, you could rewind and even pick it up again later that day or when you were in a better head space. So that's really, really beneficial for someone like me anyway.… Being online, it was just so much more achievable for me. It kind of breaks down a few boundaries. So I'd probably be more looking for something to do at home for sure.” P10
1.2 Able to practice anytime, anywhere	“It worked really well. And obviously when we went away, because we went away variably throughout the time. Then I would just use my phone or sometimes and I would do it wherever in the motel room or whatever. It was very flexible, very transportable. I was able to do it wherever I needed to.” P5
	“So my laptop was set up in its normal position, but I would unplug it and put it on the back of the couch when I went into the loungeroom. I carried it with me. That wasn't a great drama at all.” P18
	“I've got no issue with online because of the fact that there are so many things that are now online. I think it's a great option. It gives people who live in regional areas the ability to do it. It enables someone who goes on holidays to be able to do it as well. I'm absolutely glad I did it. I really am.” P20
Theme 2. Variable user experience	
2.1 Most found it enjoyable and calming	“Lots of repetition, lots of going back, and as we got more through the course, she introduced more subtlety. That's fabulous. It's really nice technically brilliant structure. Yeah. quite amazing and quite doable with her as my personal instructor effectively.” P1
	“I can't describe it. But all movements just give you some sort of relaxation, some sort of calmness. It was an immediate effect after every session of calmness, very good state of mind it put me into it.” P6
	“Just giving yourself permission to have that sort of meditative time because it's quite a relaxing state of calm I guess when you're doing Tai Chi. The pace of it, the slowness of it. So I do think that that has a flow on effect of making me calmer, just calmer in general.” P11
	“I thought the communication from the instructor was really good. She was clear and easy to understand. I thought it was thorough.” P19
2.2 Some found it repetitive and boring	“I found it really boring. I know it's slow, but I didn't expect it to be so slow, like monotonous you know.” P9
	“If it had been a week more, had it been a continuous sequence, then I would've just continued with that.” P12
	“And I don't know, the pace was very, very slow, and for a recorded thing I found it frustrating.… If it was just the recorded online, then I'd need the instructor to realize that it's a recorded medium and that we can go backwards to do things and not be quite so slow.” P17
	“I found it very slow. I am quite quick at picking things up. I found there was too much talking. Even when you were partway through a program, sometimes she'd go into that and then she would just splay the arms three times. It was like, ‘OK, I've got it. Let's just move on.’ I found that frustrating, and even by week 12 it still wasn't a full Tai Chi lesson.” P20
Theme 3. Learning challenges and strategies	
3.1 The lack of feedback can be challenging	“Whether I was doing it exactly correctly or not, I can't say. Because I had no one there to give me feedback.” P3
	“I mean when you're doing things by yourself, it just does sometimes help to be able to reach out to somebody if you've got any minor questions.” P7
	“I mean it would have been useful in the beginning to have a couple of individual sessions, particularly given that I've never done Tai Chi before.… I need someone who keeps me on the straight and narrow. If it's just left to me to do it, I'm not really good at that sometimes.” P14
	“I was thrown back into the time during COVID where we were all isolated and that sort of thing.… I really would have appreciated more of a Zoom class where there was an instructor. And even if I didn't see the other people, I could hear them or something else.” P17
	“It's not my preference. My preference is to have some personal communication and input.… Even if there was someone who you could ring if you wanted to check things.” P18
3.2 Practice makes better	“You got to really, really put in your effort to learn, especially come the difficult part. Because you practice every day there'll be definitely improvement.… Yes, definitely the more practice makes perfect. It's just that lesson you have to go back. Like cooking, baking, you need to practice more and then you'll be good at this. You had to make your time to allocate yourself to just do it. Just to do it. A lot of discipline involved.” P2
	“It's a practice. You're practicing all your life. I'll do this program again and again and again until I know this 10 Form very well because I think it needs a lot of practices to make these movements perfect how it's meant to be…. I won't be a Tai Chi master, I'm not saying that, but I think I will be better by time.” P6
	“I think that repetitive nature makes it quite easy to—I guess muscle memory and just memory to do it on your own. Good, that was easy to do.” P11
Theme 4. Online unsupervised Tai Chi is effective for most but not all	
4.1 Improved outcomes and motivated to be more active	“So the Tai Chi program definitely enhanced my physical abilities to a notable extent as well as decreasing my pain.” P1
	“I think I feel better within myself, and I also made me re‐evaluate the fact that you actually need to be healthy, as in you need to be more active. Whilst I'm not perhaps doing Tai Chi as much, then I've gone out and done other things because of this. I'm doing aqua aerobics and things like that. And I'm not sure that I would've been as motivated to do that prior to this. So I'm still feeling pretty good.” P5
	“Well, I feel confident enough to push through if I do have pain. To be conscious of the way I move as well.” P8
	“I thought I'd go back and watch the videos again. And follow along again and just park that up as a regular pattern in my week until such time when I am able to join my exercises again in a class situation.” P12
	“It probably reminded me that sort of mindfulness or that gentle breathing, those sorts of things are really important. Even just moving your body after you've been sitting at a computer and things like that. It's reminded me I've got to keep reducing the stress in stress points and moving my body a bit more.” P16
4.2 No perceived changes in outcomes	“Probably not a great deal (change) because I only definitely did go for those first two weeks fairly intensively. And then after that, practically none. I would say I didn't see a great deal of difference in how my knees were from the start to the beginning.” P7
	“Look, probably no (change). I probably didn't do it for long enough.… So nothing sort of health wise has changed, really from when I started.” P14
	“I don't think it made any difference at all. I don't think it made it worse and I don't think it made it any better, sadly. Look, I think the knowledge of the Tai Chi has been great. It was great from a meditation perspective, that slowing down. I don't know that I would do it regularly. I'd like to think I might, but I'm not sure that I would.” P20

#### Theme 1: Online unsupervised Tai Chi offers flexibility

Subtheme 1.1: The ability to pause, rewind, and repeat facilitates learning. A perceived advantage of the online Tai Chi program was the ability to pause, rewind, and repeat movements. Participants could pause the video to closely examine hand or leg positions when encountering difficult movements. Those who had attended in‐person Tai Chi group classes in the past found it to be a fast‐paced and pressurized environment where the teacher was not able to tailor classes to individual needs and abilities. On the other hand, the online prerecorded videos on the website allowed them to learn at their own pace.

Subtheme 1.2: Able to practice anytime, anywhere. Participants appreciated the flexibility of the program and being able to complete it at a time and location that suited them. Those with busy schedules could easily “slot in” a Tai Chi session within gaps in their calendars. For those living in regional areas, the online format reduced the difficulty and burden of commuting to in‐person classes. The technology was perceived to be easy to manage, allowing participants to access the program across different devices (TV, laptop, desktop, iPad, phone). This facilitated some participants to continue their Tai Chi practice while away from home (eg, while on holiday). Many participants also acknowledged that the setup to do the Tai Chi was straightforward and required minimal space. Most found it simple to clear a few chairs and create enough room at home to practice.

#### Theme 2: Variable user experience

Subtheme 2.1: Most found it enjoyable and calming. Overall, most participants enjoyed the online unsupervised Tai Chi program and felt it calmed and relaxed them. They found that each session allowed them to be present and provided “a safe space” for immersion. They also enjoyed the slow nature of Tai Chi and acknowledged the importance of repetition. In addition, most participants appreciated the clear and thorough instructions from the prerecorded videos, which made it easier to understand the Tai Chi intricacy and movement principles.

Subtheme 2.2: Some found it repetitive and boring. Some participants found the program's pace to be “too slow” and tedious and the instructions to be repetitive and boring, which led to frustration and disengagement. These individuals expressed a preference for fewer pauses and less verbal instruction in the videos. Rather than repeatedly practicing a single movement multiple times, they preferred that the instructor “skip the talking” and instead guide participants through a continuous sequence more frequently.

#### Theme 3: Learning challenges and strategies

Subtheme 3.1: The lack of feedback can be challenging. A perceived challenge with the prerecorded video format was the inability to receive feedback from an instructor or ask questions. Some participants discussed that Tai Chi involves a lot of subtlety and certain body alignments, such as pelvic movements or turns, which they found difficult to fully grasp through videos alone. They felt uncertain whether their movements were correct, reducing their confidence in completing and maintaining the program. Additionally, the unsupervised nature of the program introduced a sense of isolation and a lack of social interaction. Some participants envisaged that having someone to check in with would increase their motivation and help keep them on the “straight and narrow,” especially at the beginning or periodically throughout the program. Collectively, the absence of feedback, combined with the perceived repetitive and slow nature of Tai Chi, made the online learning experience disengaging for some participants, contributing to poor adherence for these individuals.

Subtheme 3.2: Practice makes better. It was commonly acknowledged that learning Tai Chi can be difficult and complex, especially in the beginning, due to the steep learning curve involved in understanding movement principles and mindfulness practice. However, many participants felt that more practice made learning easier. Learning strategies varied among participants, including writing down the movement sequences to memorize and practice, reading educational content, and practicing for longer periods or more frequently than the recommended three times per week. Repetition and consistency were key to learning Tai Chi, and the sense of improvement and skill mastery motivated many to continue. Many also suggested adding additional practice materials, such as refresher videos, which they could use to continue to practice and improve in Tai Chi after completing the program.

#### Theme 4: Online unsupervised Tai Chi is effective for most but not all

Subtheme 4.1: Improved outcomes and motivated to be more active. Most participants reported an improvement in their symptoms by the end of the 12‐week program. Physically, they experienced reduced knee pain, improved physical function and balance, and increased body awareness. Mentally, participants experienced more confidence in their movements, making them more willing to do activities and persevere in their daily lives despite pain. In terms of mindfulness, they found themselves better able to resettle, breathe, and calm down when faced with life's stresses, something they would not have done before this online program. Some participants also reported that they had reduced their use of pain medication. Following completion of the program, participants had varying plans. Many intended to repeat the program to maintain its benefits, whereas others wanted to try in‐person Tai Chi classes to receive some feedback. Some intended to explore other similar online Tai Chi resources due to the convenience and flexibility of the online format. Participants who were less satisfied with the online Tai Chi program reported having sought out other types of exercise, such as aerobics or yoga (either in person or online). Participants also reported that the program raised awareness and motivated them to live actively and stay “as active as possible.”

Subtheme 4.2: No perceived changes in outcomes. A few participants reported no noticeable change in their symptoms or increase in muscle strength after participating in the program. A reason suggested for this was their limited engagement, noting they had only completed a few sessions at the beginning and did not continue with the rest of the program. Their lack of adherence appeared to be influenced by low enjoyment or a mismatch between the program content and their expectations, such as anticipating a greater focus on muscle strengthening.

## DISCUSSION

Overall, most participants reported positive experiences with the 12‐week online unsupervised Tai Chi program, appreciating the program's flexibility and the ability to learn at their own pace without time or location constraints. They also noted improvements in many outcomes, including increased physical function, mindfulness, body awareness, and self‐efficacy. However, some found the program to be slow, repetitive, and boring and felt that the lack of feedback during unsupervised learning negatively impacted their adherence. To our knowledge, this qualitative study is the first to explore the experiences of individuals with knee OA in an entirely unsupervised online Tai Chi program delivered through prerecorded videos.

Consistent with findings from previous studies exploring people's experiences with Tai Chi practice,^12^, ^19^, ^37^ learning Tai Chi online in an unsupervised format was perceived to be complex, requiring repeated practice. Tai Chi not only involves physical movements but also integrates bodily awareness and sensorimotor control, requiring deliberate training and consistent practice.^12^ In our study, the instructor in the videos provided detailed instructions and repeated practice of movements, aiming to illustrate each technique and the importance of mindfulness comprehensively. Many participants appreciated the detailed instruction and felt motivated by their progressive improvement through repeated practice, which is consistent with prior studies exploring experiences with supervised Tai Chi for older people and individuals with dementia.^17^, ^38^ However, some participants found further repetitions of previously demonstrated sequences monotonous and boring. This is also consistent with qualitative findings from studies involving people with chronic back pain and cystic fibrosis who attended in‐person Tai Chi classes supplemented by home practice with DVDs.^20^, ^39^ In those studies, some found it challenging to practice on their own at home due to the boredom from repeated practice.^20^, ^39^ Collectively, this suggests that online unsupervised Tai Chi may not be acceptable to everyone and will depend on individual exercise preferences, including the importance of social interaction, past experiences, and lifestyle interests.^40^

Although the flexibility of the unsupervised and online nature of the Tai Chi program was widely recognized as a benefit,^41^ many participants perceived the lack of feedback, accountability, and social support as challenging. This finding aligns with prior studies that reported common struggles with motivation and self‐discipline during unsupervised home Tai Chi practice through DVDs.^20^, ^39^ Notably, those interventions also included in‐person classes, and it was the social interaction and peer companionship that kept participants motivated to continue practicing Tai Chi.^20^, ^39^ Our findings also broadly reflect those of a qualitative study involving people with knee OA who used an online self‐directed resistance exercise program with automated text support.^11^ This study noted a lack of human connection and a preference for in‐person care.^11^ Collectively, these findings suggest that people participating in an online unsupervised Tai Chi program compared to other forms of exercises, which are generally easier to adapt from an in‐person format to an online unsupervised format, may have a greater need for feedback, particularly those who seek human interaction. Furthermore, lack of accountability and uncertainty regarding exercise technique or execution have been found to contribute to poor adherence^42^ and may also hinder postprogram continuity.^19^ Indeed, we found that although many participants expressed an intention to continue practicing Tai Chi, few had actually continued after completing the intervention.

Our findings have implications for the design and delivery of online unsupervised physical activity programs, including Tai Chi programs. Given that some participants found the program to be boring and frustrating, it is important to signpost the time commitment and learning objectives for Tai Chi to users upfront. Allowing participants with varying experience levels to choose difficulty and progression according to their capabilities (eg, via a library of short, bite‐sized prerecorded videos, enabling users to pick and choose the most suitable options) may also help ensure that participants are appropriately challenged. Clinicians who recommend the online Tai Chi program to their patients should consider patient preferences and preferred delivery modes. Future research could explore which subgroups are more likely to benefit from unsupervised online Tai Chi to better tailor interventions and optimize outcomes. There were also perceived challenges around the lack of feedback and accountability. A hybrid model of in‐person and unsupervised online Tai Chi could provide users with initial instruction and feedback from an instructor, building their confidence for subsequent unsupervised online practice. Additional practice resources could further support continuity. There is also emerging research on technology‐enhanced online unsupervised Tai Chi showing the potential to improve the learning experience and adherence,^43^, ^44^, ^45^, ^46^ for example, incorporating digital annotations into prerecorded Tai Chi videos or using virtual reality systems to track movement patterns and generate real‐time feedback, which allows users to self‐evaluate performance without professional interference.^43^, ^44^, ^45^, ^46^

Our study has several strengths and limitations. Nesting this study within an RCT allowed for a robust evaluation of the online unsupervised Tai Chi intervention. There were several limitations. First, during the data collection, a high proportion of our participants were very satisfied with the program or had symptom improvement, so findings might be biased toward those who had more favorable experiences. To ensure analytical rigor, we actively sought and purposively sampled those who were less satisfied and those who reported no difference or worsening symptoms with treatment. When recruiting for this study, four participants reported no difference, and one participant had reported worsening symptoms in the overarching RCT (and all were included in this qualitative study). Their quotes were also incorporated into relevant themes and subthemes to capture their perspectives of the program. Second, given that the overarching RCT was unblinded, participants with a preference for online delivery or positive outcome expectations for Tai Chi may have been more likely to express interest in the study. As such, results may not be transferable to those less interested in online unsupervised Tai Chi. Third, the same researcher (SJZ) who developed the program was also the recruiter of participants in the RCT and interviewer for this qualitative study. This prior relationship with participants could have influenced results. Both coders, SJZ and TH, are physiotherapists and researchers with prior knowledge of Tai Chi as a therapeutic intervention for knee OA, and their professional and clinical backgrounds may have influenced the interpretation of the data. Although neither coder is a Tai Chi practitioner, their familiarity with the existing evidence base may have introduced a positive interpretive bias. To mitigate this, reflexivity and regular peer discussions were incorporated throughout the analysis to support rigor. In addition, a third researcher (BJL), a health scientist with no prior involvement in the development or evaluation of the program, independently reviewed all transcripts for accuracy to further minimize bias. Fourth, participants were required to own a device and access the internet, and most were highly educated (university degree or higher). This may limit the generalizability of our findings to more socioeconomically diverse or digitally excluded populations given differences in digital access and literacy, health literacy, and motivation; barriers such as time; and cultural relevance and preferences.

This study explored the experiences of people with knee OA who participated in an online unsupervised Tai Chi program. Most participants appreciated the flexibility of learning Tai Chi through this program and noted improvements in their symptoms. However, some found the program repetitive and boring and felt that the lack of feedback negatively impacted their adherence. Findings will inform necessary modifications before the planned public release of the MyJoint Tai Chi program, which aims to improve access to care and promote physical activity within the broader community.

## AUTHOR CONTRIBUTIONS

All authors contributed to at least one of the following manuscript preparation roles: conceptualization AND/OR methodology, software, investigation, formal analysis, data curation, visualization, and validation AND drafting or reviewing/editing the final draft. As corresponding author, Dr Lawford confirms that all authors have provided the final approval of the version to be published and takes responsibility for the affirmations regarding article submission (eg, not under consideration by another journal), the integrity of the data presented, and the statements regarding compliance with institutional review board/Declaration of Helsinki requirements.

## Supporting information


**Appendix S1:** Supplementary Information

**Disclosure Form**:
